# Cerebral Venous Thrombosis in the Mediterranean Area in Children

**DOI:** 10.4084/MJHID.2011.029

**Published:** 2011-07-08

**Authors:** S. Menascu, A. Lotan, B. Ben Zeev, U: Nowak-Gottl, G. Kenet

**Affiliations:** 1 Department of Pediatric Neurology, The Edmond and Lily Safra Children’s hospital, Sheba Medical Center, Tel Hashomer and the Sackler Medical School, Tel Aviv University, Israel; 2 Department of Pediatrics, E Wolfson Medical Center, Holon and the Sackler Medical School, Tel Aviv University, Israel; 3 Thrombosis Unit, The national Hemophilia center, Sheba Medical Center, Tel Hashomer and the Sackler Medical School, Tel Aviv University, Israel; 4 Universitätsklinikum Schleswig-Holstein, Rechtsfähige Anstalt des öffentlichen Rechts der Christian-Albrechts-Universität zu Kiel und der Universität zu Lübeck, Germany

## Abstract

Cerebral Venous Sinus (sinovenous) Thrombosis (CSVT) is a serious and rare disorder, increasingly recognized and diagnosed in pediatric patients. The etiology and pathophisiology has not yet been completely clarified, and unlike adults with CSVT, management in children and neonates remains controversial. However, morbidity and mortality are significant, highlighting the continued need for high-quality studies within this field. The following review will highlight aspects of CSVT in the mediteranian area in children.

## Epidemiology:

The incidence of childhood CSVT varies between 0.4 and 0.7 per 100,000 children per year.^1^–^3^ More than 40% of childhood CSVT occurs within the neonatal period, with an incidence of 2.6 per 100,000 children per year in one series.^1^ Notably, these reported figures are probably underestimates of the true rate of CSVT occurrence, since neonates often present with nonfocal neurologic signs and symptoms, potentialy leading to missed diagnosis.^2^ Old imaging techniques, the variable anatomy of sinovenous channels and rapid recanalization are all factors which may contribute to underdiagnosis.^4^ The annual incidence of sinus vein thrombosis among children in the mediteranian area is not reported. Some case- series, cohort and case controlled multicenter studies are available from Lebanon, Turkey, France, Italy, Portugal and Israel.^5^–^10^ CSVT incidence evaluated among children and neonates hospitalized in a tertiary medical center in Israel was as high as 2/10,000,^11^ potentially due to selection bias, in a referral center with high clinical awareness and high index of suspicion that led towards early diagnosis and treatment of CSVT. The largest European CSVT cohort consisted of 396 children, prospectively studied and followed in order to asses the risk factors associated with thrombosis recurrence.^12^

## Anatomy and Pathophysiology:

Cerebral vascular malformations may involve the venous circulation, with “developmental venous anomaly” (DVA) being the most frequent. This term, proposed by Lasjaunias et al.,^13^ is now widely used as a synonym for venous angioma, cerebral venous malformation, or cerebral venous medullary malformation. DVAs are encountered with an incidence of up to 2.6% in a series of 4,069 brain autopsies.^14^ They represent an anomalous venous disposition due to the absence of normal pial or subependymal veins. They may drain into both the superficial and deep venous systems, and a stenosis of the collecting vein of DVAs is commonly observed, typically at the point where the vein crosses the dura to drain into a dural venous sinus. The most frequently involved sites of CSVT are the superior sagittal sinus (90% of all cases in most studies) and transverse sinuses of the superficial venous system and the straight sinus of the deep system.^15^ After CSVT, venous pressure escalates in the occluded vessels and secondary hemorrhagic infarction develops due to brain tissue hypertension, localized venous congestion, vasogenic edema, and increased capillary hydrostatic pressure in excess of cerebral arteriolar blood flow. Approximately 65% of neonates with CSVT have brain parenchymal lesions, frequently detected in the frontal and parietal lobes.^16^

## Clinical Presentation and Symptoms:

It is apparent that the clinical manifestations of CSVT are non-specific and may be subtle. Clinical scenarios occur at all ages and the clinician should consider this diagnosis in a wide range of acute neurological presentations in childhood. These include seizures, coma, stroke, headache and increased intracranial pressure.^17^ Common illnesses, eg: ear infections, meningitis, anaemia, diabetes and head injury, may lead to CSVT evolution, various clinical settings including sepsis, dehydration, renal failure, trauma, cancer and haematological disorders may precipitate.^18^ Although presentation with pseudotumour cerebri has been well documented, there are few data on the prevalence of CSVT in otherwise unexplained hydrocephalus or in convulsive and non-convulsive seizures and status epilepticus.^19^ It has been suggested that toddlers frequently present with seizures and focal signs, mainly hemiparesis, whereas older children present with headache and changes in mental status and seizures may be less common.^20^ In a study of neonatal sinus vein thrombosis (52 neonates with a median gestational age of 39 weeks), symptoms developed at a median postnatal age of 1.5 days (range: 0 to 28 days) and consisted mainly of seizures (29 of 52).^21^ Other presenting findings in neonates were apnea (17.3%), agitation (5.8%), sepsis like(3.8%) and decreased consciousness in up to 2%.^22^ The majority of young children present acutely with seizures, focal signs and symptoms of raised intracranial pressure, such as headache and decreased level of consciousness. Subacute presentation, with chronic headache, vomiting, lethargy, anorexia or drowsiness for 3 weeks or more, may also occur.^23^

## Diagnosis and Imaging Studies:

**Figure 1:**
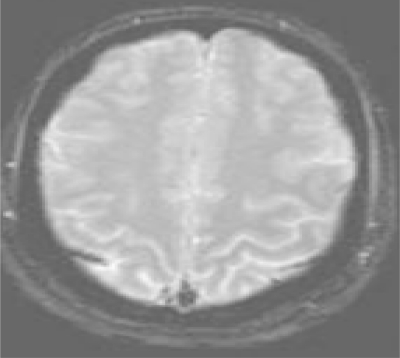
Cranial MRI- demonstrating occlusion of the sagittal sinus thrombosis.

**Figure 2:**
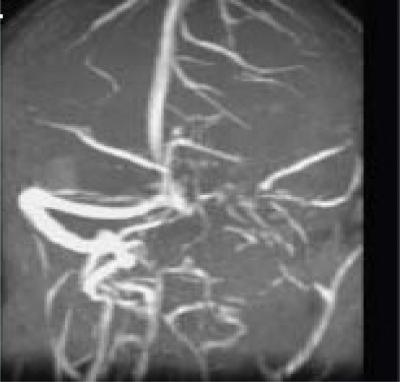
Cranial MRV- demonstrating occlusion of the left transverse and sigmoid sinuses.

The clinical spectrum of CVST closely mimics that of idiopathic intracranial hypertension. Ultrasonography, which is the preliminary screening test for hemorrhage in the neonatal period, may detect intraventricular bleeding, centrally located venous infarcts or hemorrhages, and ventricular dilation and leukomalacia secondary to CSVT infarction and ischemia. However, ultrasound has poor sensitivity for cerebral infarction and should not be used for the primary detection of CSVT and the extent of ensuing injury.^24^,^25^ Cranial computed tomography (CT) can be performed rapidly in neonates with minimum sedation but misses CSVT in 15% of cases. The classic features that indicate CSVT include the “dense triangle” or the “cord sign,” which describe the increased density over the thrombosed venous sinus in a plain CT, or the“empty triangle”[].^26^ A communicating hydrocephalus may be associated with sagittal sinus thrombosis caused by the impaired CSF absorption over the arachnoid granulations that line the sagittal sinus. A CT venogram using the multislice technique is more sensitive and specific for the diagnosis of CSVT, but the consequent radiation exposure is a definite concern in neonates. MRI is free of radiation risk and permits earlier and more accurate detection of CSVT.^27^ Diffusion-weighted imaging is a sensitive technique for detecting areas of infarction. Parenchymal changes can be seen within minutes of injury, which allows for early identification and intervention and Magnetic resonance venography allows for noninvasive assessment of the venous system without exposure to radiograph radiation. Using time-offlight techniques, the cerebral sinuses can be imaged even without the use of contrast. Currently, contrast-enhanced magnetic resonance venography, which is less susceptible to alterations in flow, is the imaging technique of choice for the detection of CSVT in the neonatal population.^28^ Attached are 2 representative MR figures of CSVT in pediatric patients (Figures 1,2).

## Etiology and risk factors:

The etiology and pathophysiology of CSVT in the pediatric population is still poorly understood, and the role of thrombophilic risk factors remains to be elucidated.^1^–^5^,^10^–^12^,^29^–^30^ Trauma, infections (especially sinusitis, mastoiditis and other conditions affecting the head and neck area), collagen vascular disorders, hemoglobinopathies, metabolic and inflamatory bowel diseases have been suggested as potential risk factors for CSVT occurrence in small cohort and case study series.^31^–^38^ Several case-control studies have dealth with the role of prothrombotic risk factors in the pathphysiology of CSVT.^1^–^5^,^10^–^12^,^39^–^44^ In a recent meta- analysis of pediatric case- controlled studies thrombophilia certainly increased the risk of stroke or CSVT in patients aged 0–18 years old, especially when combination of thrombophilic risk factors was diagnosed.^45^ A statistically significant association with first event occurrence was demonstrated for each thrombophilia trait evaluated, with no difference found between arterial ischemic stroke and CSVT. Summary ORs (fixed-effects model) were as follows: antithrombin deficiency, 7.06 (95% CI, 2.44 to 22.42); protein C deficiency, 8.76 (95% CI, 4.53 to 16.96); protein S deficiency, 3.20 (95% CI, 1.22 to 8.40), factor V G1691A, 3.26 (95% CI, 2.59 to 4.10); factor II G20210A, 2.43 (95% CI, 1.67 to 3.51); MTHFR C677T (AIS), 1.58 (95% CI, 1.20 to 2.08); antiphospholipid antibodies (AIS), 6.95 (95% CI, 3.67 to 13.14); elevated lipoprotein(a), 6.27 (95% CI, 4.52 to 8.69), and combined thrombophilias, 11.86 (95% CI, 5.93 to 23.73). Genetic thrombophilia, and especially presence of prothrombin G20210A mutation significantly increase the risk of recurrent thromboembolism in CSVT patients older than 2 years of age,^12^ in situations of future acquired hypercoagulability.

## Outcome:

Over all neonates have a more favorable outcome as compared to older children.^46^ Nevertheless, in the majority of studies the authors did not adequately describe outcomes in neonates compared with those of children with CSVT. The mean age of follow-up varied across the studies, and disabilities were variably classified.^47^ Curently, there is a substantial differences in reporting the correct neurodevelopment outcome of neonates and children with CSVT, mainly due to lack of standardized assesment protocols. Neurologic deficits including epilepsy, motor impairments, and a range of cognitive impairments were reported for 10% to 80% of all neonates on follow up. Wasay et al[]^48^ reported a higher mortality rate from CSVT in neonates, significantly associated with coma and seizures occuring at presentation. Other predictors of poor neurologic outcome were coagulation abnormalities, multiple sinus thrombosis, seizures at presentation, and venous infarction.^49^–^50^ Notably, CSVT prognosis was found to be better when compared to arterial ischemic stroke.^51^–^52^ Nearly 50% of CSVT patients do not have infarcts and may suffer fewer motor deficits or no permanent damage.^53^

## Therapy:

There are no evidence based recommendations for the therapy of thrombosis in children due to the lack of randomized controlled trials.^54^ The aim of antithrombotic therapy after CSVT is vascular patency, mitigation of thrombus growth and the avoidance of a new thrombosis in the phase of secondary prophylaxis after the acute treatment period. Therapy outcome in children differs. This is in part due to the absence of controlled trials with comparable end points. Although not treating CSVT may significantly increase the risk of thrombus propagation that is associated with new venous infarctions and worse clinical outcomes, safety issues should be considered.

Whereas in adults anticoagulant therapy of CSVT is recommended,^55^ such therapy is currently less obvious for children and neonates.^56^ A recent multi-center Portuguese study reported no influence of anticoagulation upon CSVT outcome.^57^ However, in a single center pediatric CSVT cohort, Mahendranath et. al describe a rate of 6% treatment related hemorrhage and a 31% rate of thrombus propagation in untreated patients.^58^

Notably, prospective follow up of the European pediatric CSVT cohort reflected the need for secondary anticoagulant prophylaxis in future high risk situations, especially in children with thrombophilia and lack of thrombus recanalization following acute phase therapy.^12^ Pediatric hematology expert groups recommend tailored therapy regimens based upon patient’s condition, combinations of risk factors and presumed recurrence risk.^59^–^61^

Thrombolytic therapy is generally limited to severe cases with a high risk of mortality.^62^–^64^

Generally, disease severity would be associated with antithrombotic treatment, especially in non- neonates. Markers of severity may include presentation with diffuse signs/ massive extended thrombosis or altered level of consciousness. Children with congenital or acquired heart disease require heparinization. The presence of thrombophilia and lack of transient risk factors (namely: idiopathic CSVT) determines the need for prolonged anticoagulant therapy.
